# Neuroprotective Effects of Bioactive Compounds and MAPK Pathway Modulation in “Ischemia”—Stressed PC12 Pheochromocytoma Cells

**DOI:** 10.3390/brainsci8020032

**Published:** 2018-02-08

**Authors:** Adi Lahiani, Annette Brand-Yavin, Ephraim Yavin, Philip Lazarovici

**Affiliations:** 1 School of Pharmacy, The Institute for Drug Research, The Hebrew University of Jerusalem, P.O. Box 12065, Jerusalem 9112102, Israel; adi.lahiani@mail.huji.ac.il; 2 Department of Neurobiology, The Weizmann Institute of Science, Rehovot 7610001, Israel; annette.brand@gmx.net (A.B.-Y.); ephraim.yavin@gmail.com (E.Y.)

**Keywords:** antioxidants, growth factors, ischemia, lipid peroxidation, MAP kinases, neuroprotection, NGF, oxygen-glucose deprivation and reoxygenation injury, PC12 pheochromocytoma cells, stem cells

## Abstract

This review surveys the efforts taken to investigate in vitro neuroprotective features of synthetic compounds and cell-released growth factors on PC12 clonal cell line temporarily deprived of oxygen and glucose followed by reoxygenation (OGD/R). These cells have been used previously to mimic some of the properties of in vivo brain ischemia-reperfusion-injury (IRI) and have been instrumental in identifying common mechanisms such as calcium overload, redox potential, lipid peroxidation and MAPKs modulation. In addition, they were useful for establishing the role of certain membrane penetrable cocktails of antioxidants as well as potential growth factors which may act in neuroprotection. Pharmacological mechanisms of neuroprotection addressing modulation of the MAPK cascade and increased redox potential by natural products, drugs and growth factors secreted by stem cells, in either undifferentiated or nerve growth factor-differentiated PC12 cells exposed to ischemic conditions are discussed for future prospects in neuroprotection studies.

## 1. A Brief Introduction to Ischemia

### 1.1. Stroke (Cerebrovascular Insult-CVI)

Stroke (cerebrovascular insult-CVI) is defined as a neuropathological entity which occurs when the blood flow, which supplies the brain with oxygen and essential nutrients such as glucose as well as certain bioactive molecules, is partially or entirely perturbed [1,2]. The vast majority of CVI cases are initiated by either a transient or a permanent occlusion of a major cerebral artery (i.e., “ischemic stroke”). Oxygen and glucose deprivation (OGD) in the central nervous system (CNS) can result in devastating, often irreversible consequences, eventually leading to morbidity and impaired neurological functions. The neuropathological outcome of the CVI depends on a multitude of factors such as duration and severity of the ischemia, the presence of collateral vasculature, the status of the systemic blood pressure, the etiology and localization, as well as confounding factors such as age, sex, multiple-medication and genetic background. Thus, CVI is a highly complex and heterogeneous disorder [3] which accounts for some 5.5 million human deaths annually worldwide [4]. 

Previous studies have established that at the center of the occlusion, the focal “core,” the vast majority of the cells, neurons in particular, die by necrosis [5,6] making rescue attempts almost impossible [7]. However, expansion of the damage extending beyond the core region to a greater area, also coined as “penumbra,” can lead to a secondary stage of neuronal cell death [6,8]. The reason for damage in this particular region stems paradoxically from the restoration of blood circulation (reperfusion) and resupply of oxygen and glucose. This ischemia-reperfusion-injury (IRI) process accelerates neuronal cell death through energy depletion and triggers a variety of post-ischemic responses including excessive generation of reactive oxygen species (ROS), enhanced glutamate-mediated excitotoxicity, cellular Ca^2+^ overload, lipid messenger formation through phospholipase-mediated cleavage of specific membrane phospholipids [9], ionic imbalance, neurovascular change and inflammatory processes [10]. This area of research has been central to studies developing new therapies and strategies to slow down the sequence of injurious biochemical and molecular events which eventuate in irreversible neuronal cell death [11].

### 1.2. Signaling Cascades Involved in CVI

Many studies have shown that cerebral ischemia activates in neurons a number of intricate cell-signaling cascades that are triggered by multiple lipids [12] and non-lipids [13] second messenger stimuli. Other signaling molecules are generated by a variety of non-neuronal elements such as astrocytes, microglia and brain capillary endothelial cells. These cell populations while more resistant to cell death, are nevertheless activated during ischemia by secreting various macromolecules and by perturbing the intercellular ionic balance. One such group of molecules comprises of pro-inflammatory cytokines such as IL-1 and TNF-α, which are known to initiate an inflammatory response resulting in the release of IL-6. The latter usually exhibits neurotoxic effects and may further promote ischemic injury. IL-6 can also activate phospholipase A_2_ (PLA_2_), which enhances production of inflammatory mediators such as leukotriene, prostaglandins and platelet-activating factor [14]. IL-6 and TNF-α can stimulate matrix metalloprotease (MMP) production which assists migration of leukocytes to the vascular wall and causes blood–brain barrier (BBB) impairment, leading to vascular edema and amplification of neuronal cell death [15,16]. TNF-α can also stimulate neutrophils which in the presence of Ca^2+^ give rise to superoxide anions that cause direct chromosomal and non-chromosomal DNA damage and ultimately lead to neuronal apoptosis [2]. Inflammatory cytokines also induce arachidonic acid release which, along with its eicosanoid byproducts, stimulates the release of excitatory amino acids such as glutamate to cause neurotoxicity and activate caspase-8 and caspase-3, leading to apoptosis [17]. Thus, a large profile of cellular macromolecules including proteins, nucleic acids and complex phospholipids, are actively participating in the ischemic event. The excessive presence of signaling molecules as detailed above is intimately associated with the activation of intracellular cascades which control protein phosphorylation/dephosphorylation specifically via the MAPK pathway.

#### 1.2.1. MAPK Pathway Involvement in CVI

One of the most ubiquitous players of the ischemia-triggered responses is the MAPK family of protein kinases that participates in the transduction of cellular response from extracellular to intracellular cytosolic and organelles compartments through sequential phosphorylation [18]. The activation of specific components of the MAPK cascade comprises of three, highly conserved, kinase modules consisting of MAPK, MAPK kinase (MAPKK, MKK or MEK) and MAPK kinase kinase (MAPKKK, MEKK). The MAPK family has three major members of extracellular signal-regulated kinase (ERK), p-38 and c-Jun N-terminal kinase or stress-activated protein kinases (JNK or SAPKs) which activate distinct but overlapping effector pathways [19]. These kinases are activated by phosphorylation on both threonine and tyrosine residues and subsequently phosphorylate intracellular enzymes and transcription factors. Basically, it has become evident that some MAPKs exert deleterious effects leading to acceleration of the post-ischemia neuronal apoptosis [20,21,22]. Members of the MAPK family such as JNK, p-38, have been shown to participate in the ischemia-induced neuronal damage cascade [23]. Both are activated by pro-inflammatory cytokines [24] and are believed to be mediators of cell death [25]. Activation of JNK and p-38 was also observed in vulnerable brain areas after ischemic injury [26]. However, a differential activation of members of the MAPK’s family has been observed in a CVI model in vivo [27] and after an OGD insult in vitro [28]. In those studies, MAPKs members such as ERK1/2 [27], JNK 1/2 [29] and p-38 [30] have been suggested to possess neuroprotective effects following ischemic brain injury. The protective or damaging effects on rescue or cell death by activation or suppression of MAPK members in different experimental modalities deserve careful investigations. Some are further addressed in the next paragraphs using an in vitro PC12 cell model system. 

#### 1.2.2. Antioxidants and Redox Potential Management in CVI

Antioxidants are natural or synthetic exogenous compounds or endogenous metabolites acting in the organism by several routes including removal of O_2_, scavenging of reactive oxygen species (ROS) or their precursors, by inhibiting ROS formation and by binding metal ions needed for catalysis of ROS generation. Antioxidants have been shown to act by either direct scavenging of the oxidizing radical (e.g., scavengers and chain breaking antioxidants) or indirectly as chelating agents and by regeneration of the oxidized macromolecules substrates. Both processes aim at minimizing oxidative damage.

Neurons possess a complex anti-oxidative system, consisting of various antioxidant enzymes and low molecular weight antioxidants that are widely distributed both intracellularly and extracellularly [31]. Antioxidants can be divided into two categories: one category consists of non-enzymatic bioactive compounds including glutathione, NADPH, vitamin C, vitamin E, carotenoids, uric acid and others and a second category comprising of a battery of enzyme including superoxide dismutase (SOD), glutathione peroxidase (GPX) and catalase (CAT) as well as several auxiliary enzymes. Each of the enzymes in the second category is responsible for the reduction of a different ROS and is located in a different subcellular compartment [32,33]. The distribution of antioxidants in the brain has some interesting features. Compared to other organs, there is a relatively high concentration of the water-soluble antioxidant vitamin C in the CNS unlike vitamin E, the concentration of which is not substantially different from other organs [34]. The concentration also may vary within the brain substructure regions. For instance, the lowest concentration of vitamin E is found in the cerebellum [35]. The levels of CAT are generally lower in the brain than in other tissues [34].

While the ischemic insult appears to promote upregulation of most of the defense mechanisms, that in itself appears insufficient to prevent the deleterious consequences of stroke-induced ROS generation. Indeed, a large body of evidence has attested to the fact that application of exogenous antioxidants may be effective in reducing the oxidative stress outcome [36]. 

This has been the rationale for efforts to investigate additional routes, to enhance levels of antioxidants, either by the upregulation of endogenous ones or via exogenous delivery [32]. Unfortunately, the therapeutic use of most of the latter compounds is limited since they do not cross the blood brain barrier (BBB) [37]. Although a few of them have shown limited efficacy in animal models or in small-scale clinical studies, none of the currently available antioxidants have proven to be effective in large-scale controlled studies. Therefore, any novel antioxidant molecules designed as potential neuroprotective treatment in cerebral ischemia should have the mandatory prerequisite that they can penetrate the BBB after systemic administration of cocktails containing complementary antioxidants [38] to attain an effective dose and provide an additional route for CVI therapy [37].

## 2. Experimental Models of Ischemia

The growing number of biochemical pathways involved in the pathophysiology of ischemic neuronal cell damage [39] has prompted the design of a large number of ischemic models both in vitro and in vivo. These experimental models have been instrumental to elucidate the mechanism of damage at the molecular, cellular and whole organism levels. In addition, these models were used to test potential compounds to target compromised neuronal networks [40] in order to reduce morbidity and neuronal cell damage [41]. Over the last three decades many substances of natural or synthetic origin possessing neuroprotective capacities have been studied and in some cases, even evaluated in pre-clinical studies in humans. This plethora of compounds include antioxidants [42], bioactive molecules such as chemokines [43], growth factors [44] and hormones [45], as well as drugs and steroidal and non-steroidal anti-inflammatory agents (NSAIDS) [46,47]. More recently, cellular therapies using stem cells [48] in general or mesenchymal stem cell (MSCs) in particular, have been attempted. These approaches showed some beneficial therapeutic effects which were attributed to the modulation of the systemic immune response via reduced local inflammation [49,50]. These anti-inflammatory strategies [51] acting in the production of endogenous neuroprotective bioactive molecules may be important avenues to ameliorate cerebral ischemia symptoms [52].

### 2.1. Models of In Vivo Ischemia

Induction of focal cerebral ischemia in rodents has been accomplished by a variety of surgical techniques using both endovascular and non-endovascular procedures [53]. Within the first group, the suture model has been the most widely used stroke model to study the pathophysiology of focal cerebral ischemia and evaluation of novel therapies. This model can be used to induce either a permanent or a transient occlusion of the middle cerebral artery (MCA). To achieve MCA occlusion (MCAO), a nylon filament is inserted following an arteriotomy of the common carotid artery (CCA), anterograde the site of the MCA. Cessation of the blood flow to the MCA results in reproducible infarcts within the “MCA territory.” For reperfusion, the thread is usually withdrawn usually by 90 min at the latest, after MCAO. Following the removal of the occluding element, reperfusion is restored and the animal enters a phase of ischemic-reperfusion injury (IRI). This surgical approach is relatively easy to handle, it is only moderately invasive and results in sizable and reproducible infarcts [54,55,56]. The use of macrosphere beads is another endovascular embolic stroke model resembling cardiogenic and arterio-arterial embolism which mimics the most ubiquitous etiology of stroke in humans. In this model, permanent occlusion of the MCA in rats is induced by TiO_2_-spheres [57]. Since this model allows for the remote vessel occlusion within the MRI- or PET-scanners, it permits non-invasive in vivo monitoring of both post-ischemic temporal and spatial processes in the individual animal. This model is most suitable for the preclinical evaluation of thrombolytic agents, such as the recombinant plasminogen activator [58]. The prepared thrombi (one single larger or several small ones) are injected in the internal carotid artery by an arteriotomy of the CCA, or via a stump created of the external carotid artery. A different approach to MCAO in vivo involves a sub-temporal craniotomy in order to expose the MCA. Depending on the specific method of occlusion, the MCA can then be directly obstructed by either a clip [59], an irreversible ligation [60] or cauterization [61]. Only in the former mode of obstruction a reperfusion can be permitted by removing the clip. 

Within the group of non-endovascular techniques, the application of photosensitive dyes to occlude small cortical vessels are most common [62,63]. In a typical photo-thrombosis experiment, a Rose Bengal dye solution is injected into the femoral vein, followed by illumination of the cortical surface through the intact skull bone for about 20 min, using a fiber optic cold light source with an intensity of 560 nm or laser beams. This irradiation causes a photochemical reaction of the intravascular dye, leading to local thrombosis and vascular occlusion, generating reproducible infarcts that are limited to the cortex [62,63]. 

Other modes of ischemia target directly the brain, using direct mechanical tissue insult [64], or indirectly, via whole body ischemia. The latter has also been used, particularly in newborn and young-adult animals [65] as well as on fetuses in utero [66]. In the latter approach, complete clamping of blood vessels branching from the uterine vasculature into each individual fetus has been carried out for different times of exposure during critical stages of brain ontogeny, followed by removal of the clamps to permit extended reperfusion [66]. Although all these in vivo models are presumed to replay some human conditions resembling CVI, they are technically demanding, have a low throughput and require the use of large numbers of animals for statistical consolidation [67]. 

### 2.2. Models of In Vitro Ischemia

The ability to use in vitro distinct cell populations in the CNS under culture conditions has been fundamental for advancing our understanding of ischemia. Dissociated primary cells, highly enriched in neurons or glia (oligodendrocytes and astrocytes) or mixtures of both, as well as transformed, long term and continuously propagated neuronal [68], glial [69] and oligodendroglia [70] cells have been used in the past to study cell differentiation at the structural, molecular and functional levels. The use of primary cells isolated from discrete anatomical and functional brain structures to study molecular, cellular and generation of functional networks in a well-controlled and reproducible environment constitutes still the highlight of the in vitro culture approach. Yet to this stage, culture of pure cell populations particularly of neuronal origin, is still technically complex, with great variabilities from laboratory to laboratory and usually is of low yields. A different approach to address some of the disadvantages of dissociated primary cultures, consist of using organotypic brain slices with limited life span ex vivo [71] or neuronal stem cells [72] and neurospheres [73]. These relatively long-term cell cultures offer the possibility to accomplish a high-throughput analysis, which becomes relevant while testing novel, potentially neuroprotective compounds [4].

However, for all practical purposes, to overcome the caveats of the primary cell culture system, clonal cell lines derived from neuronal and glial tumors and immortalized in vitro, have been established. These cell lines have the advantage of being able to grow fairly easy under reproducible conditions to enable unlimited cell harvest and minimal variability between cell batches. They are also transferable from one laboratory to another. It is necessary to apprehend that due to their multiple origins they may express distinct physiological differences based on the cell type they were originally derived from. Transformed secondary cell lines have been obtained from murine and human neuroblastoma and retinoblastoma [74,75], pheochromocytoma [68,76,77], glioma [76] and oligodendroglia [70] tumors. The immortalized cells can grow indefinitely until certain stimuli applied to allow cell differentiation and acquisition of functional properties. Many of these clonal lines may exhibit an advanced neuronal phenotype by manipulating culture conditions, such as serum deprivation, addition of cell cycle inhibitors, specific growth factors or compounds such as cyclic AMP. 

In vitro models have been widely used in furthering our understanding of ischemia, by studying individual pathological pathways and molecular components comprising the ischemic cascade [78] and by identification of unique cellular signals involved in neuroprotection. In order to enhance the reliability and reproducibility of hypoxic/ischemic in vitro conditions using neuronal cell cultures, rigid and validated OGD/R insults protocols are necessary. At this stage, there is a great diversity between individual experimenters with respect to good laboratory practice (GLP) which needs attention, particularly for generalized conclusions. For example, there is a wide range of techniques used for generating hypoxic/anoxic conditions including anaerobic incubators [79], hypoxia workstations [80] hypoxic chamber glove boxes [81] or homemade ischemic devices [82,83]. To that, one should add the variability in oxygen replacement by argon, nitrogen or carbon dioxide. Argon belongs to the group of noble gases, which are regarded as chemically inert. Astonishingly, argon exerts neuroprotective effects [84]. Therefore, mixtures of N_2_/CO_2_ are mostly employed to regulate O_2_ levels. Furthermore, there is the issue of the mode of nutrients and growth factors deprivation, which deserves consideration. Needless to say, in vitro systems cannot reproduce the full constellation of changes which occur during cerebral ischemia in vivo. Indeed, the term ‘ischemia’ has little meaning in a model system lacking blood flow. For assessment of neuroprotective potency of various agents, it is deemed to examine the effects of oxygen and glucose deprivation with or without reoxygenation (reperfusion), which is compromised in vitro substituting for the pathological events occurring during in vivo ischemia. There is no practical way to completely remove oxygen from growth media used in open culture systems. Even if the cell culture medium is thoroughly deoxygenated, this situation ends the moment the ischemic device is exposed to air (for example, to exchange culture medium or adding a neuroprotective compound). There are two general approaches for exposing in vitro neuronal cultures to anoxic or hypoxic conditions. For experiments performed under the microscope or for electrophysiological recordings using brain slices, unique chambers are used which allow rapid perfusion with deoxygenated solutions [85]. In these experiments, the brain slice thickness is small enough to allow residual oxygen to be rapidly metabolized by the brain tissue itself. Achieving fully anoxic conditions is more difficult for dissociated neuronal cells or transformed neuronal cell cultures since even with rapid perfusion of anoxic medium and with anoxic gas flowing over the cultures, oxygen tension is not reduced below 15–30 mm Hg [86]. Anoxia experiments are better performed if cells are placed in a sealed ischemic chamber [82]. To achieve this goal, many investigators have used a sealed plastic container with shelves for culture dishes or plates, which is flushed with an anoxic gas and then placed in a temperature-controlled incubator. This method is straightforward but the severity of anoxic exposure is influenced by the residual oxygen in the culture medium. Using small volume of cell culture medium, oxygen was detectable in the medium for more than 60 min despite rapid perfusion of anaerobic gas [87]. Therefore, the duration of true anoxic (0% O_2_) or hypoxic (1–20% O_2_) conditions depends not only on the time of incubation in the chamber but also on the rate of diffusion of oxygen out of the medium and on the rate of consumption of oxygen by cultured cells. This presents a serious potential obstacle, as oxygen utilization is likely to be significantly influenced by culture density, medium volume and by the experimental neuroprotective compounds under study. For these reasons, a system in which the cultured neuronal cells are washed into pre-deoxygenated medium in a hermetically-closed chamber, is highly recommended. In this way, the duration and intensity of anoxia or hypoxia remain under stringent experimental control [77]. The duration and intensity of ischemic injury are critical parameters in evaluation of neuroprotective potential drugs. Insults causing 40–60% cell death are optimal for investigations of putative neuroprotective compounds; however, at higher cell death values it is difficult to achieve significant neuroprotection [88,89,90]. Therefore, it is very important to establish insult duration intensity–neuroprotection relationship using cells treated with a bioactive compound before progressing to its tedious characterization in the in vivo ischemic model [91,92]. A necessary control in OGD/R insult experiments are cells exposed to the compound under study using normoxic (22% O_2_) conditions to allow basal cell death estimation. The neuroprotective effect, defined as the percent decrease in cell death in the presence of an investigational neuroprotective compound upon subtracting basal normoxic levels, is normalized to untreated OGD/R cultures. Calculation of a neuroprotection index, defined as the fractional ratio of cell death in treated versus control neuronal cells is a useful index to establish the rescue efficacy of the neuroprotective compound for comparison between the different analogues [88]. 

#### PC12 Cells as a Representative In Vitro Ischemic Catecholaminergic Neuronal Model

The clonal line PC12 originally derived from a solid rat adrenal medulla tumor has been widely used as a dopaminergic neuronal model for in vitro studies of neuronal cell differentiation [93]. During the proliferative stage, the cells are rounded with diameters ranging between 6 and 14 μm, have a doubling time between 48 and 96 h and are considered catecholaminergic due to their ability to synthesize and release catecholamines [94]. They also transport catecholamines via amine-transporters into chromaffin type granules and actively release them, in response to cytosolic Ca^2+^ elevations [95,96]. 

When exposed to nerve growth factor (NGF), PC12 cell division is halted, a neurite network commences and cells become electrically excitable acquiring properties of an adrenergic neuronal phenotype. As such they resemble mature sympathetic neurons [97]. These changes are accompanied by a series of biochemical transformations that take place at all subcellular levels including plasma membrane, cytosol, cytoskeletal network and nucleus. Some of these events are independent of transcription, while others clearly involve changes in gene expression [98]. PC12 cells serve as a principal dopaminergic model in molecular neuroscience for investigating NGF mechanisms of action under normal or after various insults [99]. In addition, the ability to grow PC12 cells in continuous culture with a well-defined secretory cell phenotype has been advantageous for studying secretory pathway mechanisms. 

In addition to unraveling aspects of differentiation into neuronal phenotypes, PC12 cells have been an excellent in vitro tool to investigate certain aspects of various neurological disorders such as glutamate excitotoxicity [100], Parkinson- disease [101], Alzheimer disease [102] and epilepsy [103] and are instrumental for studying oxidative-stress related consequences on neuronal cell survival [104]. Studies using PC12 cells also addressed issues such as the impact of serum-starvation [105], NGF- deprivation [106] and drug cytotoxicity [107]. 

In our own studies a typical oxygen-glucose-deprivation (OGD) modality was attained in PC12 cells using a growth medium that had been completely or partially deprived of glucose and oxygen [89]. The restoration of normoxic oxygen and glucose, also denoted as reperfusion, has been another essential paradigm that needs appropriate attention. The ischemic-PC12 neuronal model [77,108] mimics in part stroke pathology since it is composed of a two-phase strategy; an OGD insult and a reperfusion (R) period which addresses the restoration of oxygen to the insulted tissue. Whereas drug-induced neuroprotection is mostly effective upon pre-treatment of the cultures before OGD [109], investigation and development of alternative methods for cell-induced neuroprotection during the R phase [77] would be more practical and relevant for clinical use. Under these “ischemic” conditions, the insulted PC12 cells were found to release large amounts of lactate dehydrogenase (LDH) and prostaglandin PGE_2_ and to reduce ATP levels. The stressed cells exhibit signs of apoptotic cell death as emphasized by a decrease of the BAX protein in the cytosol, release of cytochrome C from the mitochondria, activation of caspase-3, reduction of cyclin D1, an increased redox potential, DNA fragmentation and differential activation of MAPKs [77,82,110,111]. Compared to in vivo ischemic models, the PC12 cultures as well as other neuronal in vitro models require a longer episode of energy depletion to induce cell death. Typically, the time window of OGD sufficient to induce widespread neuronal apo-necrotic cell death in PC12 cells and brain primary neuronal cultures ranges between 1 and 4 h, while glial cultures require 6–24 h, [78,110]. The advantage of a catecholaminergic phenotype displaying characteristics of both adrenal chromaffin cells and sympathetic neurons [93] render the PC12 cells useful for further exploring neuroprotection and mechanisms of action of neuroprotective drugs [110] as detailed below. 

A big disadvantage of using dissociated primary neurons derived from embryonic CNS tissue is the fact that upon terminal differentiation, cells can no longer be propagated. Unlike primary neurons, transformed neuronal-like cells can be used in vitro indefinitely to overcome this limitation. The major catecholaminergic cell lines include the neuroblastoma SH-SY5Y cells derived from a human tumor origin [112,113,114] and the Neuro-2a (N_2_A) cells derived from a rodent [74,75]. In general, these cells lines of neuroepithelial origin, are highly proliferating [115,116] but can be induced to differentiate by a variety of means including serum withdrawal, or addition of dibutyril cAMP, NGF or retinoic acid resulting in adrenergic or cholinergic phenotypes [116,117,118,119]. The N_2_A cell line has been originally derived from a spontaneous mouse neural-crest derived C-1300 tumor and used extensively to study neuronal differentiation, axonal growth and signaling pathways [116]. Another caveat requiring attention is the fact that SH-SY5Y cells contain two morphologically distinct: neuroblast-like and epithelial-like phenotypes. The former phenotype expressing neuroblast-like morphology tests positive for tyrosine hydroxylase and dopamine-β-hydroxylase catecholaminergic markers, whereas the latter is devoid of these enzymatic activities [120]. They can be also differentiated to a more mature neuron-like phenotype that is characterized by several neuronal markers [121]. However, few studies have addressed the biological significance of the adherent versus floating phenotypes but most studies utilize adherent populations and discard the floating cells during media changes [121]. 

Many catecholaminergic cell lines have been used to study OGD/R neurotoxicity in vitro [122] and they appear to respond differently to neurotoxic insults [122,123]. We choose to focus on the PC12 cells, a clonal line that has been widely characterized vis a vis molecular, cellular, functional and stress studies [77,98,99] to the point of being used to test validation of many compounds used in clinical therapy [88,90,110]. The cell line is available from the ATCC repository– PC-12 (ATCC^®^ CRL-1721™). 

## 3. Chemicals and Cell-Induced Neuroprotection in PC12 OGD/R Model

### 3.1. Drugs and Natural Products

The diversity and number of biochemical pathways associated with stress and oxidative stress in particular, has prompted comprehensive investigations on a large number of potential neuroprotective compounds using neuronal cultures to elucidate their course of action. These neurotherapeutic approaches consist largely of induction of natural, endogenous antioxidants via the enzymatic cellular machinery [110] or by cell culture media supplemented with antioxidants [42] or synthetic drugs [34]. 

In Table 1 a compendium of selected molecules conferring neuroprotection using the PC12 cell model and the OGD reperfusion (OGD/R) insult is presented. For the sake of brevity, several stressors such as chemical hypoxia (CoCl_2_, cobalt chloride) or exposure to free radical donors (H_2_O_2,_ sodium nitroprusside) were not included. The stress paradigm includes only O_2_-deficiency and glucose deprivation. The methods used to estimate cell death and/or neuroprotection are also indicated in Table 1. Considering that the type and severity of the ischemic insult may significantly affect the neuroprotective outcome, particular attention was given to the type of insult (hypoxic or anoxic and with or without hypoglycemia) and the duration of both ischemic and reoxygenation episodes. Needless to say, an undifferentiated or NGF-differentiated phenotype of the PC12 cells may also affect the outcome and therefore, it was carefully addressed in the studies described in Table 1. 

Table 1 depicts a wide and diverse collection of bioactive substances which include various drugs, opiates, antibiotics, anticoagulants, antioxidants and Ca^2+^ channel blockers of distinct chemical composition. A common denominator to all these compounds is their ability to confer a certain degree of neuroprotection when added to stressed PC12 cells. This characteristic feature appears independent of the duration of the OGD/R (IRI) insult and is effective in either undifferentiated or NGF-differentiated cells. This pattern consists of an overall general reduction of Ca^2+^ overload and a reduced redox potential as reflected by a reduced lipid peroxidation (LPO). 

A second interesting observation is an apparent common trait of the differential modulation of the MAPK family. In this respect, the PC12 cells provide a clear example of how the canonical signaling MAPK cascades can elicit diverse processes such as neuritogenesis, gene induction and cell proliferation [34]. Notably, the MAPK’s family is differentially activated during both IRI in vivo [140] and also during the OGD insult in vitro [111]. Inhibition of ERK1/2 [140], JNK 1/2 [141] and p-38 [142] by a variety of agents, including low molecular weight, natural and/or synthetic antioxidants has been suggested to possess beneficial (neuroprotective) effects following ischemic brain injury [143]. Table 1 indicates that part of the neuroprotective effect of certain drugs such as Ca^2+^ channel blockers and anti-coagulants is mediated by an attenuation of excessive Ca^2+^ influx, a primary event in the excitotoxicity cascade during IRI in one hand, and/or inhibition of MAPK’s stress kinases such as Jnk and p-38, on the other hand [142]. Other studies indicate a mechanism of action that includes reduction of the redox potential, causing a decrease of reactive oxygen species (ROS) and lipid peroxidation. While the direct correlation between chemical-induced neuroprotective effect and inhibition of ischemia-induced stress kinases JNK1/2 and p-38 α/β phosphorylation activity is expected, the effect on MAPK isoforms is more complex. Compounds such as naloxone and antioxidants such as saponins are characterized by a positive correlation between phosphorylation of ERK and induction of neuroprotection. Others, such as minocycline and homocarnosine are characterized by a negative correlation between inhibition of phosphorylation of ERK and induction of neuroprotection. These results may be explained by the complex physiological role of ERK under normal as opposed to stress, pathophysiological conditions [21]. Thus, on the one hand, MAPKs are key signaling enzyme which, by their phosphorylated substrates, regulate a variety of cellular essential functions such as survival, growth, proliferation and differentiation. On the other hand, MAPK isoforms can also activate apoptotic cell death pathways following aberrant pathological conditions such as oxidative stress [21]. The molecular basis for this functional dual activity which regulate MAPK members in general and ERK isoform activity in particular, under physiological or stress conditions (i.e., IRI-OGD/R) remain unclear and deem further investigation. Recent findings indicating Activin A/Smads differential inhibition of MAPKs [144] and the apparent correlation between miR-494-mediated modulation of Sox8 and MAPK [145] may provide a promising line of investigation to resolve this puzzle.

Table 1 also depicts a group of compounds classified as thiol and NO donors which are active during redox processes. α-Lipoic acid (α-LA, 1, 2-dithiolane-3-pentanoic acid) is one of the most potent bioactive natural thiol antioxidants and a well-established ROS scavenger [27]. It is a low molecular weight compound taken up by the gastro-intestinal system and once in the circulation, can cross the BBB. In brain cells, α-LA is reduced to dihydrolipoic acid (DHLA) [28] which is subsequently exported to the extracellular medium; hence, protection is provided to both intracellular and extracellular compartments. α-LA has been shown to be a key antioxidant, to regenerate through redox cycling with other antioxidants such as vitamin C, as well as to raise intracellular glutathione levels. Given its clinical safety, [29], α-LA is considered a potential molecule for CVI treatment. Early studies in both humans and experimental animal models found that α-LA can decrease redox potential markers of oxidative stress following stroke or traumatic brain damage [27,30]. Administration of α-LA to rodents has shown to reduce damage that occurs after ischemia–reperfusion injuries in the central [146] and peripheral nervous system [147]. α-LA has been found to prevent hydrogen peroxide-induced neuronal damage [148], protect neurons from neurotoxicity in Parkinson’s disease [149] and reduce oxidative damage following stroke by enhancing the levels of superoxide dismutase type 2 (SOD_2_) [146]. α-LA is approved as a drug against diabetes comorbidities and since 1966 is available by prescription [150].

The second compound in this group (Table 1) Tempol (4-hydroxy 2,2,6,6,-tetramethylpiperidine-1-oxyl) is also a powerful potential antioxidant. It is a non-toxic synthetic nitroxide compound with antioxidant properties [151] which originally has been used for magnetic resonance imaging in humans [152]. It possesses a superoxide dismutase (SOD)-like activity and has been shown to modulate nitric oxide (NO), reduce metal levels and react with peroxyl and carbonyl-centered radicals, as well as dismutate oxygen radicals [153]. Tempol can restore its own antioxidant capacity [153]. Its ability to cross the cell membrane [154] provides a higher potency for intracellular activity, in contrast to other antioxidants that fail to effectively penetrate the cell membrane. Tempol has been shown to confer neuroprotection in a Parkinson’s disease model [155] and to attenuate cocaine-induced cell death through decreased oxidative damage [156] in PC12 cells. In a mouse model of traumatic brain injury, Tempol protected brain tissue from ischemic damage [157] and was neuroprotective in a rat model of stroke and transient focal ischemia [158]. Currently, Tempol is used in the clinic as a topical drug to prevent radiation-induced alopecia [159]. 

In our studies, we have linked the α-LA and Tempol antioxidants via a polyethylene glycol (PEG) bridge and generated AD3, a novel synthetic bifunctional antioxidant (Table 1) [88]. Using this conjugate, we found that it conferred a remarkable neuroprotection capability in OGD/R-treated PC12 cells [88]. A 2–3-fold enhanced protection was noticed in PC12 cells compared to the precursor moieties, indicating an intrinsic potent neuroprotective activity generated. Further experiments revealed that MAPK phosphorylation was substantially reduced. This was accompanied by marked changes of the redox potential of the cells. Since oxygen and nitrogen radical species are known to be responsible for the activation of MAPKs [160], we raised the possibility that by scavenging these radicals (i.e., decreased redox potential and lipid peroxidation), AD3 indirectly suppressed the excess activation of MAPKs, thus conveying neuroprotection [88]. 

Previous studies have argued that mixing various doses of complementary antioxidants [38] may bring about a greater neuroprotection efficacy. AD3, in contrast, suggests that linking the two antioxidants molecules into one synthetic bifunctional conjugate, may present a more potent neuroprotective approach than a mixture of both [88]. Such bifunctional strategies were designed previously, to generate successful co-drugs such as l-Dopa-glutathione [161], l-Dopa-carnosine [162], LA-ibuprofen [163] and Tempo-NSAIDS [164]. These co-drugs have shown a remarkable bifunctional free radical scavenging activity and as a result have been used for the treatment of Parkinson’s disease and certain inflammatory diseases. Further in vivo studies characterizing the efficacy and BBB penetrability of AD3 are required in order to clarify its potential for brain IRI therapy.

### 3.2. Mesenchymal Stem Cells: A Prospective New Tool in Neuroprotection

Stem cells are pluripotent cells which have the ability to renew and give rise to a wide range of mature cell types in the human body under appropriate conditions [165]. Stem cells can be derived from embryonic, fetal and adult tissues and generally classified into three groups, embryonic stem cells (ESC), somatic stem cells/adult stem cells (SSC/ASC) and reprogrammed stem cells (iPSC) [166]. Given their proliferation and differentiation capacities, stem cells have great potential for the development of novel cell-based therapies [167,168]. Although ESC and fetal stem cells are considered an attractive source for therapy of experimental stroke, their application is limited due to restricted availability, formation of teratomas and ethical concerns [169,170]. In contrast, bone marrow, cord blood and placental stem cell and derived progenitors have been shown to be efficient in the therapy of many diseases of the CNS, including stroke [171]. Very few studies suggest that the therapeutic effect of transplanted stem cells is associated with their ability to generate new graft-derived neurons and glial cells [172] as a cell replacement therapy. Most studies will agree however, that stem cells possess the capability to secrete different proteins, including growth factors, cytokines, chemokines, metabolites and bioactive lipids, which have paracrine and autocrine therapeutics activities. Therefore, ASC cells have been thoroughly studied upon transplantation in experimental CNS diseases [173] and their neuroprotective potential after cerebral ischemia has been repeatedly demonstrated in vivo [171,174,175,176] as well as in vitro ischemic models [48,175]. 

Mesenchymal stromal stem cells (MSCs) are spindle shaped plastic-adherent cells that have been intensively studied for their multipotent differentiation ability in vitro [177,178] MSCs have the potential to differentiate into osteoblasts, chondrocytes, adipocytes, hepatocytes and neurons [179]. They can be found and isolated from many different mesenchymal tissues of the body, including bone marrow, placenta and adipose tissue [180,181]. The International Society for Stem Cell Research has defined MSCs as plastic adherent cells with an attached fibroblast-like morphology in standard conditions which can be differentiated into adipocytes, chondrocytes and osteoblasts under in vitro differentiating conditions. In addition, while they should express mesenchymal markers such as CD105, CD90 and CD73 they should not express hematopoietic markers as CD45, CD34, CD14, CD79a, CD11b and HLA-DR [182]. 

PLX cells are MSCs derived from human placenta, which were expanded in a validated, pharmaceutical bioreactor. PLX are adherent cells, characterized by highly selective expression of typical markers of MSC such as CD73, CD90 and CD29 and at extremely low levels of hematopoietic (CD45, CD34), endothelial (CD31) or dendritic cells markers [183]. A comparison between the surface markers of PLX cells to bone marrow-derived MSCs strongly indicates that PLX possess a MSC-like phenotype. It has been demonstrated that PLX cells are a promising source of cells for therapy of ischemic disorders in a rat model [183] and in critical limb ischemia generated in mice and known in humans [184]. Their therapeutic effect was evaluated, at low and high doses, in several ischemic disorders in human clinical trials for peripheral artery disease, regeneration of injured gluteal musculature after total hip arthroplasty and for treatment of intermittent claudication (http://www.clinicaltrials.gov), due to their immunomodulatory beneficial therapeutic effect [183]. PLX cells have been shown to secrete a variety of protective anti-inflammatory cytokines and growth factors and also to exert a protective capacity in vivo in stroke therapy and in traumatic brain injury [185,186,187].

Table 2 summarizes the recent evidence that IL-6 and VEGF165 are secreted by MSC from a variety of tissue sources and their potential role in neuroprotection, both in rat or rabbit stroke in vivo models, as well as in primary neurons, neuroblastoma and PC12 cells OGD/R in vitro models. Secretion of IL-6 appears beneficial in diminishing cell death in a wide spectrum of cytotoxic events generated by serum deprivation or by treatment with toxic substances such as calcium ionophore [188], 6-hydroxydopamine, 4-hydroxynonenal [189,190], MPP+ tetrahydro-isoquinoline [191] and hydrogen sulfide [192]. VEGF_165_ is a major angiogenic factor that has been known for its neurotropic activity in PC12 cells [193] after exposure to beta-amyloid [194] and glutamate [195] or subjected to hypoxia [196]. VEGF_165_ tested in an OGD insult in primary cultures of rat neurons also exhibited neuroprotective properties [197,198]. It was also effective in vivo in a rat model of cerebral ischemia [199]. 

Table 2 provides some common features of neuroprotection associated with addition of these bioactive macromolecules using diverse endovascular stroke models in vivo, as well as employing primary cortical neurons and neuroblastoma cells exposed to in vitro OGD models. It has been shown that these compounds secreted by MSC cultures, activate the MAPK pathway leading both to transcription-dependent and -independent events. A large variety of trophic factors including VEGF and IL-6 have been detected and isolated from secreted proteomes derived from human and rat MSC tissues including bone marrow, placenta umbilical cord stroma, adipose tissue and cornea. 

In addition to those trophic factors, MSCs were also found to secrete neurotropic factors such as brain-derived neurotrophic factor (BDNF), basic fibroblast growth factor (bFGF, FGF-2), neurotrophin-3 (NT-3), glial derived neurotrophic factor (GDNF), transforming growth factor β (TGF-β), interleukin 4 and 10 (IL-4, IL-10), etc. It is proposed that these neurotrophic factors are responsible for the neuroprotection measured in vivo [211,212]. MSC therapy improves outcome of stroke mainly by secreting paracrine factors. Although MSCs are able to cross the BBB [213], those that are transplanted by direct intracerebral injection or by intravenous routes, appear to selectively migrate to the ischemia boundary site [214]. Given the relatively small number of MSC cells arriving to the injured brain site, the secretion of bioactive macromolecules by the MSC cells appears to amplify production of endogenous neurotrophic factors by stimulating endogenous neurogenesis and by affecting neuro inflammatory processes, to enhance neuroprotection [211]. This suggests that providing the therapeutic molecules that are secreted by these cells (bystander effect) could be another effective neuroprotective strategy [215]. This paracrine cell-induced neuroprotective effect promotes novel pharmacological understanding for clinical therapy in ischemic disorders by using the secretome of the transplanted MSCs [173].

### 3.3. Effect of Growth Factors on PC12 Cell Rescue after OGD/R Insult

The neuroprotective effect and proposed mechanisms of action of a diverse group of growth factors on OGD/R-challenged PC12 cells is summarized in Table 3. 

Typical neural related growth factors such as NGF, as well as atypical ones such as Insulin-like growth factor (IGF-1), fibroblast growth factor (FGF-10), Activin A of the TGF-β superfamily, Erythropoietin (EPO) and Heparin-binding epidermal growth factor like (HB-EGF) are listed. 

A common characteristic shared by these growth factors is that activation of their respective tyrosine kinase (TRK) receptors initiates an intracellular signaling cascade via Ras activation and other down-stream members of the MAPK family [77,111,216,217]. Signals from this physiological pathway ultimately modulate gene expression which is responsible for cell survival and differentiation [218,219]. Activation of TRK receptors by these growth factors also involve changes in intracellular Ca^2+^ (PLC γ pathway; [220] PI_3_K (Akt pathway; [221]) and the overall cellular redox potential [222,223] contributing to neuroprotection.

Activation by growth factors is usually accomplished by phosphorylation of distinct proteins which, via transcription-dependent or-independent mechanisms, activate survival pathways and confer neuroprotection. To date more than 160 cellular substrates of ERK phosphorylation have been discovered, all of which are expressing a multitude of functions [228]. PC12 cells for example, were shown to possess substrates such as hypoxia inducible factor 1 (HIF_1_α) [229], nuclear factor-erythroid 2 p45-related factor 2 (Nrf_2_) [230], the human heat shock protein 70 (HSP 70) [229], other protein kinase and phosphatases such as Rho kinase [231], protein phosphatase 1/2 A (PP-1/2A) [232], cytoskeletal proteins (Cofilin) [233] and different regulator proteins of apoptosis (caspase-3/9) [108]. Table 3 also demonstrates that, independent of insult duration, or time of reperfusion these seemingly distinct growth factors, exert a substantial degree of protection as attested by a significant reduction of apo-necrotic cell death. 

**Figure 1:**
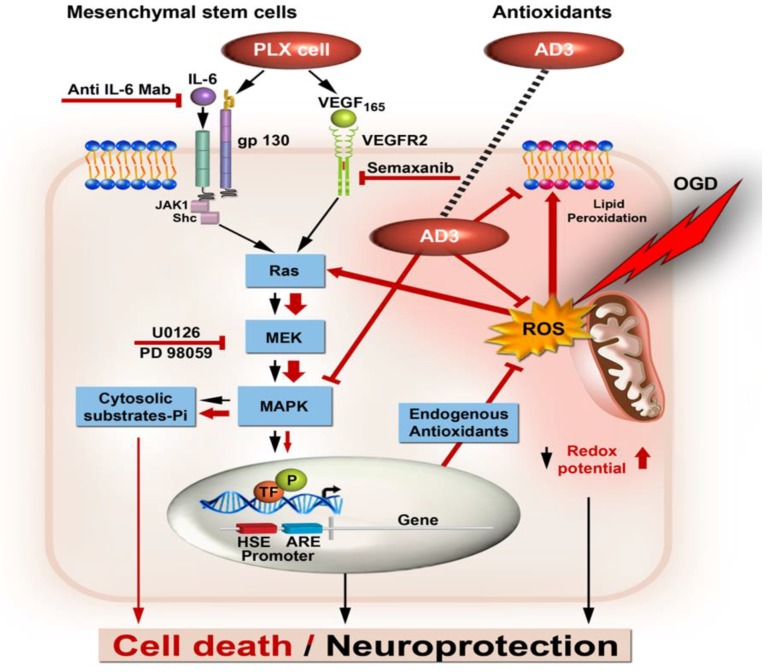
Schematic representation of convergent pathways of neuroprotection generalized for growth factors secreted by mesenchymal stem cells and synthetic antioxidants. Black arrows—physiological pathways; Red arrows—pathological pathways (wider arrow-hyperactivation); Red lighting—oxygen glucose deprivation (OGD); Red bars—inhibitory effect. ARE, antioxidant response element; gp130, glycoprotein 130; HSE, hypoxia response element; IL-6, interleukin 6; Mab, monoclonal antibody; P, Phosphorylated; ROS, radical oxygen species; TF, transcription factor; VEGF, vascular endothelial growth factor.

A schematic representation of cell death/cell rescue following treatment with growth factors and/or antioxidants under ischemic conditions is illustrated in Figure 1.

In this diagram, PC12 cells were stressed by an OGD/R insult and as a result excess ROS species generated activates via Ras the MAP kinase cascade and its ubiquitous MAPK’s representative ERK [234,235]. The excessive ROS production and differential pathological hyperactivation (Figure 1, wider red arrow) of MAPK members such as ERK isoforms [77,111] leads to cytosolic protein substrates phosphorylation and is initiating the apoptotic cell death cascade (Figure 1, red arrows) expressed by caspase-3 activation, cytochrome C leakage from the mitochondria, BAX cellular redistribution, cleavage of poly (ADP-ribose) polymerase (PARP) and DNA fragmentation [108]. Therefore, it is suggested that the degree of phosphorylation of the different MAPK members ultimately determines the subsequent fate of the cell [236,237,238,239]. Neuroprotection provided by exogenous supplements under those circumstances, can be envisaged by several possible mechanisms; first, exogenous antioxidants such as α-LA and Tempol may reduce directly the levels of the ROS. Second, AD3 in addition to reducing the levels of ROS, also decreased by 20–50% stress-induced MAPK member’s phosphorylation activities. Indeed, AD3 action is strongly associated with the MAPK pathway as attested by the U0126 specific MAPK inhibition [88]. AD3 might act directly on phosphorylated ERK levels or most probably indirectly, by enhancing ERK-dependent, specific transcription factors-induced, nuclear promoter activation, as previously reported for anthraquinones and flavones-induced neuroprotection [230,240]. Activation of the latter post translational machinery may enhance the battery of intracellular anti-oxidative enzymes [241] which, in turn, may affect the overall redox potential conferring neuroprotection. 

The action of the growth factors IL6 and VEGF secreted by PLX cells comprise a second group of bioactive molecules discussed above, which act mostly under physiological conditions (Figure 1, black arrows). IL6 and VEGF released by PLX cells, also act on the MAPK cascade via Ras activation. Under those circumstances ROS-induced activation of tyrosine kinase receptors (RTKs) [242] leads to stimulation of the MAPK pathway [243]. Alternatively, ERK1/2 activation is seconded by ROS-induced oxidation and Ras activation [235]. In either scenario, ERK activation is likely to phosphorylate transcription factors (TF, Figure 1) such as Nrf_2_, a transcription factor known to regulate gene expression and others that are controlling the levels of antioxidant enzymes such as Heme oxygenase 1 (HO-1) and NAD(P)H: Quinone oxidoreductase 1 (NQO-1). The latter gene products will act to deplete intracellular ROS level and as a result confer neuroprotection [241]. Using Affymetrix GeneChip^®^ Rat Genome 230 2.0 Array (Thermo Fisher Scientific, Waltham, MA, USA), we have shown that following OGD/R stress, many genes containing “Antioxidants Responsive Elements (ARE)” are stimulated as compared to control, normoxic conditions [77]. Among the most overexpressed genes found were Aldolase (2.2 fold induction), HO-1 (4.9 fold), Phospho-fructokinase (1.7 fold), Enolase (1.5 fold), Sugar transporter superfamily, Oxidoreductase, Homologous to global ischemia-induced gene 11 (Ero 1L, 1.7 fold) and Hypoxia induced gene 1 (HIG1-1.5 fold). Moreover, HO-1 was further induced by the neurotrophin NGF that conferred neuroprotection towards ischemic insults in the PC12 cell model [77]. Therefore, the induction of these genes appears to be a stress-response of the cells to combat the consequences of the ischemic insult. In this context, the remarkable assistance to reduce cell death exerted by the AD3 antioxidant [88] supplements can be viewed as an external help to provide the system with sufficient antioxidant levels. 

## 4. Some Lessons from the PC12 OGD/R Model

Ischemic brain injury is a complex sequel of events which can result in irreversible damage in areas immediate to the site of injury as well as further away due to lack of rapid intervention. The vulnerable areas, also commonly known as penumbra, constitute the ultimate target for rescue once the occlusion has occurred. These areas are characterized biochemically and pathologically by two molecular events which appear to govern the fate of the compromised tissue i.e., a marked depletion of antioxidant levels, resulting from tissue reoxygenation and the activation of intracellular signaling cascades which are involving MAPK family members. 

Administration of bioactive substances to the compromised sites for reducing the consequences in the penumbra area should consist of either suitable BBB-penetrable antioxidant cocktails or growth factors supplements that act via well-established membrane receptors. These bioactive substances should control the overall cellular redox potential and turn on essential intracellular signaling cascades such as those controlled by the MAPK family which are beneficial for cell neuroprotection. 

The lesson learned from the PC12 cell model of OGD/R stress calls for two types of bioactive molecules, antioxidants and growth factors, as suitable candidates with a potential for applicability to human CVI pathology. One type concerns the use of antioxidants, which exert protection on cellular macromolecules (nucleic acids, proteins and membrane lipids) from free radical damage. In this context, the conclusion from this review would be to call for a strategy of well-designed antioxidant cocktails of bifunctional compounds (i.e., AD3) which show high BBB permeability, sustained activity and entrance into the cell. Improvement of the molecular design of such bioactive compounds may assist in reducing the size of the deprived penumbra area and open novel therapeutic possibilities. Secondly, use of suitable growth factors to suppress augmentation of damage via modulation of intracellular pathways is another strategy for cell rescue and neuroprotection. This modality for future intervention is more complex, as it is necessary to develop better techniques for long term growth factors selective release in the CNS, as achieved today by transplantation of MSC. The OGD/R model has shown the potential of growth factors and antioxidants to boost nuclear factors as a means to improve neuronal cell rescue. 

A focus on molecular and pharmacological aspects by which bioactive molecules and stem cell therapy confers neuroprotection in IRI experimental models will become increasingly important for drug discovery. The PC12 OGD/R pharmacological model is an excellent, validated model system to study cellular and molecular mechanisms of neuronal death and to reveal molecular and pharmacological modalities to enhance neuroprotection. However, even within this in vitro experimental model, there are substantial variables which need standardization. Unfortunately, with respect to translation into therapy, many of the compounds tested in clinical trials have not undergone vigorous and adequate pre-clinical testing using in vitro or in vivo ischemic models. The lack of translational success of any neuroprotective molecule could be attributed to a number of causes but many of these, we would argue, originate from methodological drawbacks. Thus, a complete picture as to whether a particular compound may fulfill its potential of providing a neuroprotective effect for ischemic stroke in the clinic remains to be rigorously examined. Some differences between pre-clinical studies and clinical trials in assessing efficacy for neuroprotective agents include: therapeutic time window, dose, type and insult duration, primary cell death end point, etc. In addition, confounding biochemical parameters such as ATP levels, cell redox potential, MAPKs activities, lipid peroxidation and others, should be closely monitored in order to assess if a candidate compound is producing neuroprotection by modulating these parameters. These differences need to be considered when designing pre-clinical studies. Addressing these caveats will more closely align pre-clinical studies to clinical trials and may improve the chances of successful translation for neuroprotection. In order for these neuroprotective drugs to succeed, essential quality standards need to be adhered to; however, these must remain realistic as the evidence that standardization of procedures improves translational success remains absent for stroke [244]. It is also proposed that more sophisticated clinical outcome neuroprotection scales characterizing neurological deficits in animal models and stroke patients will allow for better evaluation of treatments translated from the laboratory. Although past trials of neuroprotective agents in ischemic stroke indicated limited success, significant research insights into mechanisms of stroke, ischemic models and trial design have incrementally improved approaches for future therapies. The use of neuroprotective agents in combination with existing thrombolytic therapy might be applied in clinical settings and is expected to be greatly beneficial for early intervention in stroke.

## Figures and Tables

**Table 1:** Neuroprotective effect and proposed mechanism of different drugs and natural products using PC12 hypoxic and ischemic model.

Drug Classification/Name	Insult Oxygen: O_2_/CO_2_/N_2_ % Glucose: +/−	OGD/Reoxygenation Duration (h/h) ^a^	Differentiation by NGF (Days) ^b^	Neuroprotections Assays ^c^	Mechanism ^d^	Reference
**Calcium Channel Blockers:**					Reduced Ca overload and redox potential	[90,124,125]
Nimodipine and Nifedipine	1/5/94, −	18	Yes (10–14)	LDH, Caspase 3 activity, MMP, MTT, Hoechst/PI		
Mebudipine	0/5/95, −	0.5–1.5/24	No			
Cinepazide maleate	0/5/95, −	2.5/24	No			
**Anti-coagulants**	0/5/95, −			MTT, Annexin-PI, MMP	Reduced Ca overload and redox potential	[126,127]
Heparin		4	No			
Benzopyrone derivative		24	No			
**Opiates**	0/5/95, −	24	No	LDH	Enhanced activation of ERK1/2	[128]
Naloxone						
**Tetracycline antibiotic**	1/5/94, −	Hypoxia 2–4	No	MTT	Reduced activation of ERK1/2 and p-38	[129]
Minocycline						
**Brain natural histidine dipeptide antioxidants**	0/5/95, −	24	No	LDH, Caspase 3 activity	Reduced activation of ERK1/2, JNK1/2 and p-38	[108]
Homocarnosine-antioxidant						
**Saponins**				Annexin-PPI, LDH, Tunnel, MTT		[130,131]
Ginsenosides	0/5/95, −	6	No		Enhanced activation of ERK1/2	
Dioscin	1/5/94, −	24	No		Reduced activation of ERK1/2, p-38, JNK	
**Sesquiterpene alkaloid**	0/5/95, −	24	No	LDH, Hoechst, MTT, DNA fragmentation	Reduced redox potential and LPO	[132,133]
Huperzine A acetylcholinesterase inhibitor						
**Stilbenoid Phenol antioxidant**	1/5/94, −	24	Yes (>7); No	Annexin, Caspase 3,9 activity, MTT, Hoechst/PI, LDH	Reduced redox potential, LPO and Ca overload, Increased antioxidant level	[134,135,136]
Resveratrol						
**Glycosyl anthraquinone antioxidant**	0/5/95, -	24	No	LDH, Annexin V, MMP, MTT	Reduced redox potential and LPO	[137]
Aloin						
**Thiol antioxidants**	0/5/95, −	24	Yes (5)	LDH, MMP, Caspase activity, MTT	Reduced redox potential, Ca overload and activation of p-38	[138]
s-Methyl cysteine (SMC)						
**Thiol and NO donors antioxidants**	0/5/95, −	18	No	LDH	Reduced redox potential, LPO and activation of ERK1/2, p-38, JNK	[88]
α-Lipoic Acid						
Tempol						
α-Tempol ester-ω-lipo ester PEG-AD3						
**Lipid metabolism co-factors**	0/5/95, −	0.5–2/4	No	DNA fragmentation, TUNEL, MTT	Reduced redox potential and LPO	[139]
l-carnitine , Acetyl-l-carnitine						

^a^ Duration of oxygen and glucose deprivation (OGD) and Reoxygenation insults. ^b^ No—undifferentiated PC12 cultures; Yes—PC12 culture treated with NGF for different days as mentioned in the parentheses. ^c^ Neuroprotections assays: Necrosis: LDH (lactate dehydrogenase); Apoptosis: Annexin V, MMP (mitochondrial membrane potential), Caspase 3,9 activity, DNA fragmentation, TUNEL assay; Viability: MTT (3-(4,5-Dimethylthiazol-2-yl))-2,5-Diphenyltetrazolium bromide, Hoechst/PI. ^d^ Ca—calcium; LPO—lipid peroxidation.

**Table 2:** Involvement of VEGF and IL-6 in neuroprotective effects of mesenchymal stem cell.

Species	Mesenchymal Stem Cells Origin	Ischemic Insult, Duration (h) and Model	Cytokines Estimation	Neuroprotection Evaluation	Mechanisms	Reference
Human	Bone marrow	Rat MCAO 1 h	Gene expression	Neurological score	Increased expression of rat and human VEGF, IL-6 AND rat bFGF, BDNF, NT-3, GDNF, CCL2	[200,201,202]
		Human M17 neuroblastoma cell line-OGD (20–24/72)	Cytometric Bead Array	Infarct volume		
	Umbilical Cord stroma	Mice MCAO 1.5–2 h	ELISA	Neurological score	Increased secretion of human VEGF_165_, upregulation of IL-4, TGF-β1 and IL-10	[2,51,203,204]
		Mice primary cortical neurons- OGD/R (2/24)	Gene expression	Infarct volume	Decrease secretion of IL-6, IL-1β	
		Rat MCAO 2 h	IHC, Angiogenic Microarray			
		Rabbit MCAO 2 h	Western blot			
	Placenta and umbilical cord blood	NGF differentiated PC12	Gene expression	Necrosis—LDH	Increased secretion of human VEGF and IL-6,	[89,92]
		(7 days)—OGD/R (4/18)	ELISA	Neuroprotection Index	Reduced redox potential and LPO	
	Adipose tissue	Rat MCAO 1 h	IHC	Neurological score	Increased expression of human VEGF	[205,206]
		Phase II clinical trials		Infarct volume		
		Patients with acute ischemic stroke within 24 h of onset	Not applicable	NIH Stroke scale/score (NIHSS)		
	Cornea	Rat MCAO	ELISA, Cytokine array, Gene expression	Infarct volume Open field memory test	Increased expression of human VEGF and BDNF	[207]
Rat	Bone marrow	Rat MCAO 1 h	ELISA, Western Blot, IHC	Neurological score	Increase expression of VEGF and bFGF	[208,209,210]
				Infarct volume		
	Adipose tissue	Rat MCAO 1 h	IHC	Neurological score	Increased secretion of rat VEGF	[205]
				Infarct volume		

**Table 3:** Neuroprotective effect and proposed mechanism of different growth factors using PC12 OGD/R model.

Drug Classification/Name	Insult Oxygen: O_2_/CO_2_/N_2_ % Glucose: +/−	OGD/Reoxygenation Duration (h/h) ^a^	Differentiation by NGF (Days) ^b^	Neuroprotections Assays ^c^	Mechanism ^d^	Reference
Nerve growth factor (NGF)	0/5/95, −	18	No	LDH, caspase activity	Inhibition of JNK 1, p-38 α, p-38 β activity	[77,111]
Insulin-like growth factor-1 (IGF-1)	0.1/5/94.9, −	18	No	MTT	ERK1/2 and PI3K pathways contribute to neuroprotection	[216]
Fibroblast growth factor 10 (FGF-10)	1/5/94, −	2–4/0	No	LDH, Annexin	Attenuation of redox potential	[224]
TGF-β superfamily (Activin A)	0/5/95, −	3–16/0	Yes (3)	Caspase-3, MTT	Modulation of gene expression	[225]
	0/5/95, −	6/0	Yes (6)	Annexin, Caspase-3, MTT	Attenuation of redox potential; Modulation of cellular endogenous antioxidants	[226]
	0/5/95, −	1–24/0	No/Yes (7)	Morphological changes, Annexin, MTT	Modulation of gene expression	[218,219]
Erythropoietin (EPO)	1/5/94, −	18	Yes (5)	Pro-apoptotic gene, MMP	Attenuation of redox potential	[227]
Heparin-binding epidermal growth factor like (HB-EGF)	0/5/95, −	21	No	LDH, Annexin, MTT	Activation of ERK1/2 phosphorylation	[217]

^a^ Duration of oxygen and glucose deprivation (OGD) and Reoxygenation insults. ^b^ No—undifferentiated PC12 cultures; Yes—PC12 culture treated with NGF for different days as mentioned in the parentheses. ^c^ Neuroprotections assays: Necrosis: LDH (lactate dehydrogenase); Apoptosis: Annexin V, MMP (mitochondrial membrane potential), Caspase 3,9 activity, DNA fragmentation, TUNEL assay; Viability: MTT (3-(4,5-Dimethylthiazol-2-yl))-2,5-Diphenyltetrazolium bromide, Hoechst/ PI. ^d^ Ca—calcium; LPO—lipid peroxidation.

## Abbreviations

AD3	A PEG conjugate of Lipoic acid with Tempol
BDNF	brain-derived neurotrophic factor
b-FGF	Basic fibroblast growth factor
CCL2	chemokine (C-C motif) ligand 2
CVI	cerebrovascular insult
ELISA	enzyme-linked immunoassays
ERK	extracellular signal-regulated kinase
GDNF	glial cell derived neurotrophic factor
IHC	Immunohistochemistry
IL-6	interleukin-6
MAPK	mitogen activated protein kinase
MCAO	Middle Cerebral Artery Occlusion
MSC	mesenchymal stem cells
NGF	nerve growth factor
NT-3	neurotrophin-3
OGD	oxygen and glucose deprivation
OGD-R	oxygen glucose deprivation followed by reoxygenation
TGF-β1	Transforming growth factor beta 1
VEGF	vascular endothelial growth factor
